# Study of the D-dimer, C-reactive protein, and autoantibodies markers among HBV infected patients in Babylon province, Iraq

**DOI:** 10.37796/2211-8039.1186

**Published:** 2021-12-01

**Authors:** Ahmed Abdul-Abbas Bayram, Hussein O.M. Al-Dahmoshi, Noor S.K. Al-Khafaji, Raheem Tuama Obayes Al Mammori, Ali Husain Shilib Al-Shimmery, Morteza Saki

**Affiliations:** a Babylon GIT Center, Babylon, Hilla City, Iraq; b Department of Biology, College of Science, University of Babylon, Babylon, Hilla City, Iraq; c Department of Microbiology, Faculty of Medicine, Ahvaz Jundishapur University of Medical Sciences, Ahvaz, Iran

**Keywords:** ANA, CRP, D-dimer, dsDNA, HBV, Iraq

## Abstract

**Background:**

Hepatitis B can be defined as one of the dangerous diseases caused by the hepatitis B virus (HBV), which infects the liver and causes liver failure, cirrhosis, and death.

**Aims:**

This study aimed to evaluate the D-dimer, C-reactive protein (CRP), and autoantibodies markers among HBV-infected patients in Babylon province, Iraq, compared to a healthy control group.

**Methods:**

In this cross-sectional study, all patients referred to GIT and liver centers in Merjan Medical City, Babylon, Iraq from January 2016 to January 2018 were screened for HBV infection by quantitative real-time polymerase chain reaction (qPCR). Antinuclear antibody (ANA), dsDNA, D-dimer, and CRP markers were examined using fluorescence technique in randomly selected patients and some healthy individuals as control group. Statistical analysis was performed using SPSS program.

**Results:**

In this study, 424 HBV patients from different areas of Babylon province, Iraq were enrolled. A total of 40 patients and 15 healthy participants were selected for evaluation of D-dimer, CRP, ANA, and dsDNA levels. The results of the distribution of HBV-infected patient by gender per area revealed that males accounted for a higher percentage than females in all parts of Babylon provinces. Also, a highly significant increase in serum levels was observed in the patients compared to the control subjects for all the studied parameters D-dimer, ANA, dsDNA, and CRP. Overall, 5.5% of HBV patients (3/40) had a positive ANA titer. None of the HBV patients had a positive dsDNA titer.

**Conclusion:**

This study showed that, HBV-infected patients had elevated levels of D-dimer and CRP compared to the control group. Also, the seropositivity titers of ANA and anti-dsDNA autoantibodies were low in Iraqi HBV patients.

## 1. Introduction

Hepatitis can be defined as an inflammation of the liver, caused mainly by physical damage, alcohol, drugs, viral infections, toxins, and autoimmune reactions [1,2]. Hepatitis B can be defined as one of the dangerous diseases caused by hepatitis B virus (HBV) that infects the liver and causes liver failure, cirrhosis (liver scarring), lifelong infections, liver cancer, and death. In addition, vaccination against hepatitis B is important for any contact with a patient and for all newborns. Vaccinations are also required for all medical and nursing personnel. In most cases, specific treatment is not required for acute hepatitis B [3–5].

Worldwide, approximately 257 million people are chronically infected with HBV and require treatment [6]. Because they must be followed for many months or years, such treatment can be expensive. Also, treatment of the chronic hepatitis B requires consistent medical examinations as well as monitoring to evaluate the progression of the virus by determining viral load and DNA copy number every 3 months during the treatment protocol. Some individuals who have HBV do not fully recover from the infection (chronic infection). These individuals carry the virus and could infect others in the remaining years of their lives [7,8]. Iraq is considered a country with intermediate endemicity for HBV, as shown by the 3% seroprevalence of HBsAg in the normal population [9]. Also, the country is considered to have low endemicity for HBV compared to its neighboring countries. The increase in prevalence of all types of hepatitis in Iraq could be due to the overcrowding of migrants and refugees and security situation [10]. Numerous studies on HBV in various regions of Iraq, have shown that HBV infection ranges from 0.65% to 3.3% [11–17].

D-dimer can be defined as a specific metabolite related to cross-linked fibrin formed by fibrinolytic enzymes in plasma. Plasma D-dimer could be referred as one of the additional prognostic new markers for one-month mortality in HBV-related decompensated cirrhosis (HBV-DeCi) [18]. Elevated D-dimers have been associated with poor prognosis in cirrhotic patients and may serve as one of the new markers to predict short-term mortality in patients with acute-on-chronic liver syndrome [19,20].

C-reactive protein (CRP) is synthesized by hepatocytes during acute inflammatory and infectious processes as part of the host innate immune response. CRP levels can increase within a short period of time in response to an acute stimulus such as infection, and this increase can reach 1000 fold of normal value [21]. Furthermore, there was a strong correlation between the ability of the virus to cause liver damage and elevated serum CRP concentrations [22]. Anti-double stranded DNA antibody (anti-dsDNA) and antinuclear antibody (ANA) facilitate the evaluation and diagnosis of patients with various systemic auto-immune conditions. ANA can be found in the chronic infectious diseases [23,24]. Although many studies have indicated autoantibody positivity in patients with chronic hepatitis C, the literature rarely reports this positivity in patients with chronic HBV. Since no preliminary study has been conducted on the mentioned factors in patients with HBV in Babylon city, this study aimed to investigate the dsDNA, CRP, D-dimer, and ANA levels in HBV- infected patients compared to a healthy control group from aforesaid city in Iraq.

## 2. Materials and methods

This research was approved by the Scientific and Ethical Committee of College of Science, University of Babylon, Hilla City, Iraq following the Declaration of Helsinki. Verbal informed consent was obtained from all participating individuals. This cross-sectional study was performed from January 2016 to January 2018, with 424 HBV patients (290 in 2016, and 134 in 2017) enrolled in the center GIT from different areas of Babylon province, Iraq. A total of 40 patients were selected for evaluation of D-dimer, CRP, ANA, and dsDNA levels using standardized manual principle and immunofluorescence procedure of Biomed Company. Considering the prevalence of 0.65%–3.3% of HBV infection in various regions of Iraq and using the following formula, the sample size in this study was set to 40: $n=\frac{z^{2}p(1-p)}{d^{2}}$. In this formula, the different factors were used as follows: z = 1.96, p: 0.027, and d = 0.05. Also, 15 apparently healthy individuals were studied as a control group. The selection of participants in the control group was based on the negative tests for all HBV-associated seropositivity markers in their serum. All selected patients had normal liver enzyme levels: serum alanine aminotransferase (ALT), aspartate aminotransferase (AST), prothrombin time (PTT), and total serum protein and albumin. Basic diagnosis and viral load were determined with the Sacace quantitative real time polymerase chain reaction (qPCR) kit (Sacace/Italy) using Smart Cycler (Cepheid/USA) (cut-off value, 25 copies/ml equals 6 IU/ml). The qPCR technique was used in all hepatitis-positive patients in the follow-up and treatment program with a combination of interferon-alpha 2b and oral tenofovir drugs. All hepatitis patients admitted to the Babylon GIT center go through the following processes, recording and documentation with clinical information for each patient. Viral screening tests, routine biochemical and hematological investigations, autoantibody profiles for liver diseases were done to rule out the autoimmune hepatitis (such as ANA, ASMA, LKM, SLP, and p-ANCA). Statistical analysis of data was performed as ANOVA, Chi-squire, and Pearson correlation using SPSS program. The level of *p* < 0.05 was considered significant.

## 3. Results

**Fig. 1:**
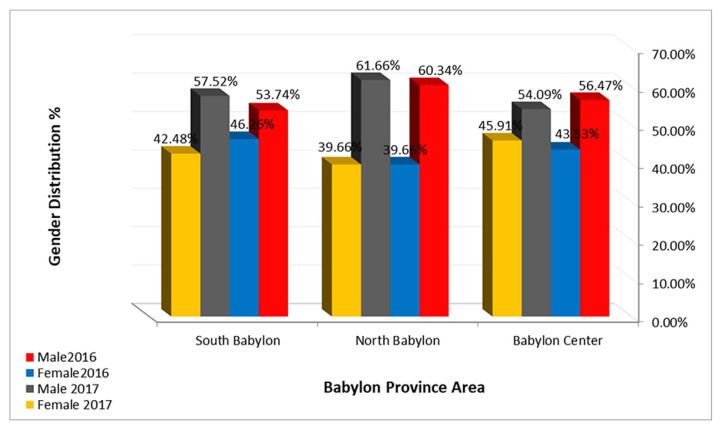
Geographical and gender distribution of HBV infected patients of Babylon province.

The demographic characteristics of the patients studied are shown in Table 1. The geographical distribution of the recorded patient cases registered at routine admission in GIT Disease Center in the present work is shown in Fig. 1. The results show that the distribution of all recorded cases was different in various areas of Babylon province. Most HBV infections occurred in the northern areas, while a lower percentage was recorded in the southern part of the province. The male patients were mostly infected with HBV than the females as shown in Fig. 1 of all the recorded cases. The percentage of male patients was higher than female patients in all areas of Babylon province.

From all the recorded patients, 40 patient samples were selected to determine certain parameters compared to 15 samples of healthy participants as controls. The results showed a highly significant increase in D-dimer and CRP in the patients compared to the control group, while the result of ANA and dsDNA antibodies showed no significant change: D-dimer level (mean ± SD = 1006.12 ± 190.0) in patients compared to (171.33 ± 93.6) in control group; ANA (86.87±34.67) in patients compared to (55.33 ±9.34) in control group; CRP titer in patients (13.96 ± 11.39) compared to (3.06 ± 1.30) in control group; and dsDNA in patients (75.12 ± 14.25) compared to (53.0 ± 11.30) in control group (Table 2).

The results also showed that 5.5% (3/40) of the patient samples had positive ANA titers. ANA-positive patients had lower D-dimer titers compared to ANA-negative patients and controls (Table 3). CRP levels were also higher compared to ANA-negative patients and controls. The major titer level of ANA-positive patients was 200 IU/ml compared to 77.7 IU/ml in negative patients and 55.3 IU/ml in control group. Only 5.5% (3/40) of all collected samples were ANA-positive, while no dsDNA positive samples were detected. The ANA-positive patients had lower D-dimer level (429.4 ± 31.48) and higher CRP levels (23.7 ± 19.97).

Regarding the gender distribution of the studied parameters (Table 4), 60% of the patients were male and 40% were female. The D-dimer level was greatly reduced in male patients compared to females, while no significant differences were found in CRP, ANA, and dsDNA between male and female patients.

**Fig. 2:**
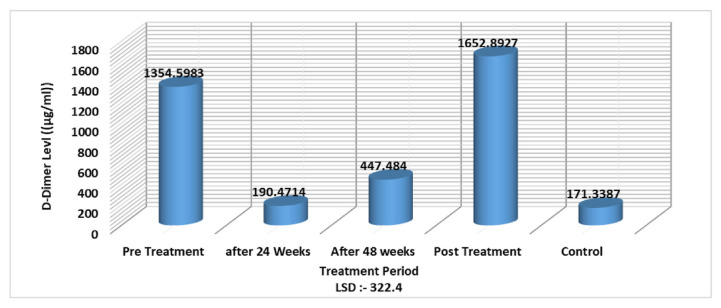
D-dimer level in association with treatment period of HBV infected patients.

Also, the results showed that the D-dimer level increased in hepatitis patients before and after treatment, while there were no significant differences in patients during the treatment period at 24 and 48 weeks (LSD value 322.4). These results may relate to the effect of treatment on liver tissue, by inhibiting fibrinolysis factors, resulting in a decrease in D-dimer levels during these treatment periods (Fig. 2).

**Fig. 3:**
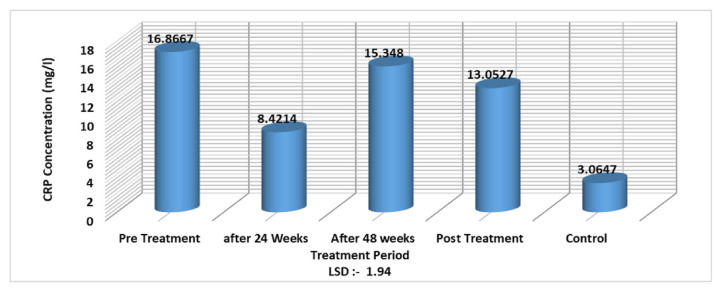
CRP level in association with treatment period of HBV infected patients with interferon-alpha 2b and oral tenofovir drugs.

As for CRP level and treatment, the results showed a significant decrease in CRP level only after 24 weeks with an LSD value of −1.94 (Fig. 3).

**Fig. 4:**
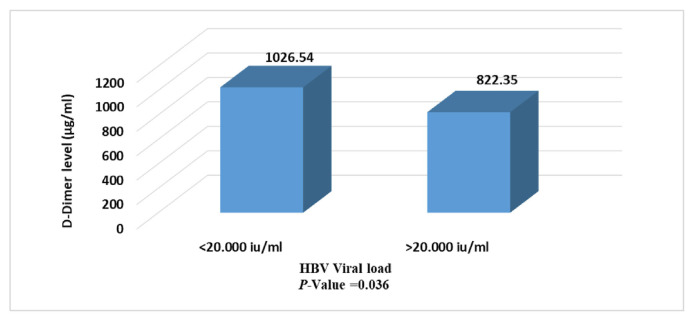
D-dimer level in association with viral load in HBV infected patients.

Figure 4 shows significant differences in D-dimer level related to HBV viral load as patients with viral load less than 20.000 IU/ml had higher level of this marker than patients with >20.000 IU/ml ( *p* = 0.003).

**Fig. 5:**
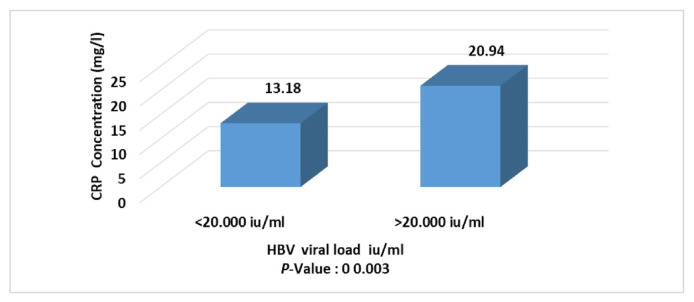
CRP level in association with viral load concentration in HBV patients.

The result in Fig. 5 shows that CRP level is higher in patients with elevated viral load (more than 20.000 IU/ml) than in patients with low viral load (less than 20.000 IU/ml).

## 4. Discussion

In this study the higher percentage of infections in the northern region might be due to the large number of population, crowded conditions, popularity of cupping therapy shops, prevalence of some religious traditions, and low health education among the people. Blood cupping (hijama) poses a significant transmission risk for HCV and HBV [25–27]. Increasing tattooing has also been implicated as an HBV transmission route in urban centers [28–30]. Also, the proportion of male patients was higher than that of female patients in all areas of Babylon province. These results were consistent with various Iraqi researches that found a higher prevalence of HBV in males compared to females [11–17].

The results showed a highly significant increase in D-dimer and CRP in patients compared to control samples, while ANA and dsDNA antibodies showed no significant change. These results reflect that disease activity could lead to an increase in D-dimer and acute phase reactants such as CRP. Monitoring of disease prognosis can be done using such parameters as a non-specific signpost to show the poor or good prognosis of viral infections and liver tissue damage. The liver is considered the production site for most proteins that promote or inhibit fibrinolysis and coagulation. In addition to high CRP levels, D-dimer could serve as a new biomarker for acute-onchronic liver failure (ACLF) syndrome and to evaluate deterioration and short-term mortality in patients with liver failure due to HBV [20,31].

In this study, the CRP level showed a significant decrease at weeks 24 with an LSD value of −1.94 (Fig. 3) after treatment with interferon-alpha 2b and oral tenofovir drugs. The results of the current study were in accordance with the previous study by Huang et al. [32] that CRP levels were high before the treatment with interferon-alpha/antiviral and decrease directly after treatment. The decreased CRP level suggests that the systemic inflammatory responses in HBV infection could be effectively alleviated after antiviral therapy [32]. CRP levels in individuals treated with antiviral therapy were low compared to untreated individuals, which may indicate an improvement in inflammation [33,34]. Another finding of the current study was the higher CRP level in patients with increased viral load (more than 20.000 IU/ml) compared to patients with low viral load (less than 20.000 IU/ml). Recently, some data suggested that CRP may be one of the prognostic factors for cirrhosis and cancer [35,36]. Elevated CRP concentrations at baseline have been associated with subsequent liver cancer incidence and mortality in chronic liver disease. The results of this work suggest that systemic inflammation may serve as one of the long-term markers of liver disease and liver cancer [36]. Also, the immune system produces more acute phase proteins in response to viral disease. If the number of viruses or viral activity is increased, this could lead to high production of acute phase proteins such as CRP.

In this study, no HBV-infected patients had a positive dsDNA antibody titer, while a study by Acay et al. [37] from Turkey found a seropositivity rate of 1.5% (1/67) in patients with chronic hepatitis B. Also, in their study, ANA was positive in 12% of the chronic HBV patients, which was higher than the current research. In another report from Turkey by Şener et al. [38], 17% (8/47) of HBV patients had an ANA positive titer, which was higher than our result. Although positive autoantibody titer may occur in inflammatory autoimmune diseases, some reports have shown this positivity in non-autoimmune diseases including infectious diseases such as HCV and HBV infections [39,40].

## 5. Conclusions

This study showed that HBV-infected patients had elevated levels of D-dimer and CRP compared to the control group. Also, the seropositivity titer of ANA and anti-dsDNA autoantibodies was low in the Iraqi HBV patients.

## Figures and Tables

**Table 1:** The demographic characteristics of studied patients.

**Age groups (years)**
1–10 [n =1 (2.5%)]
11–20 [n =4 (10%)]
21–30 [n =5 (12.5%)]
31–40 [n =7 (17.5%)]
41–50 [n =8 (20%)]
51–60 [n =7 (17.5%)]
61–70 [n =6 (15%)]
>70 [n =2 (5%)]
**Gender**
Males [n =24 (60%)]
Females [n =16 (40%)]
**Accommodation**
Urban [n =15 (37.5%)]
Rural [n =25 (62.5%)]

**Table 2:** Concentration of All studied parameters among HBV infected patients compared to control.

Descriptive		N	Mean	Std. Deviation	*P*-Value
D-dimer (μg/ml)	Patients	40	1006.12	190. 2	0.009
	Control	15	171.33	93.61	
ANA Titer (index)	Patients	40	86.87	34.67	0.060
	Control	15	55.33	9.34	
dsDNA Titer (index)	Patients	40	75.12	14.25	0.071
	Control	15	53.00	11.30	
CRP level (mg/l)	Patients	40	13.96	11.39	0.000
	Control	15	3.06	1.30	

**Table 3:** Association between ANA positive patients with all studied parameters.

ANA and studied parameters		N	Percentage	Mean	Std. Deviation	*P*-Value
D-dimer (μg/ml)	Positive	3	5.5	429.4	31.48	0.021
	Negative	37	67.3	1052.8	197.09	
	Control	15	27.3	171.3	93.61	
ANA Titer (index)	Positive	3	5.5	200.0	34.64	0.000
	Negative	37	67.3	77.70	9.09	
	Control	15	27.3	55.3	9.34	
dsDNA Titer (index)	Positive	3	5.5	56.6	20.20	0.000
	Negative	37	67.3	76.6	12.91	
	Control	15	27.3	53.0	11.31	
CRP level (mg/l)	Positive	3	5.5	23.7	19.97	0.001
	Negative	37	67.3	13.2	10.47	
	Control	15	27.3	3.1	1.31	

**Table 4:** Association between all studied parameters and gender of patients.

Studied parameters	Gender	N	Percentage	Mean	Std. Deviation	*P*-Value
D-dimer (μg/ml)	Male	24	60	638.7	144.6	0.015
	Female	16	40	1557.1	237.9	
ANA Titer (index)	Male	24	60	86.4	30.5	0.075
	Female	16	40	87.5	41.1	
dsDNA Titer (index)	Male	24	60	75.2	16.2	0.095
	Female	16	40	75.0	11.1	
CRP level (mg/l)	Male	24	60	13.8	10.3	0.063
	Female	16	40	14.2	13.2	
